# *Lacanobia oleracea nucleopolyhedrovirus* (LaolNPV): A new European species of alphabaculovirus with a narrow host range

**DOI:** 10.1371/journal.pone.0176171

**Published:** 2017-04-20

**Authors:** Oihane Simón, Martin A. Erlandson, Marie Frayssinet, Trevor Williams, David A. Theilmann, Anne-Nathalie Volkoff, Primitivo Caballero

**Affiliations:** 1 Bioinsecticidas Microbianos, Instituto de Agrobiotecnología, CSIC-UPNA, Gobierno de Navarra, Mutilva Baja, Navarra, Spain; 2 Sakatoon Research and Development Centre, Agriculture and Agri-Food Canada, Saskatoon, Saskatchewan, Canada; 3 UMR 1333 INRA—Université Montpellier 2, Diversité Génomique et Interactions Microorganismes-Hotes (DGIMI), Montpellier, France; 4 Instituto de Ecología AC, Xalapa, Veracruz, Mexico; 5 Summerland Research and Development Centre, Agriculture and Agri-Food Canada, Summerland, British Columbia, Canada; 6 Dpto. Producción Agraria, Universidad Pública de Navarra, Campus Arrosadía s/n, Pamplona, Spain; Wuhan Bioengineering Institute, CHINA

## Abstract

During an insect sampling program in alfalfa crops near Montpellier, France in 2011, *Lacanobia oleracea* larvae were collected that died due to nucleopolyhedrovirus infection (LaolNPV). This virus was subjected to molecular and biological characterization. The virus was a multiple nucleocapsid NPV that showed similar restriction profiles to *Mamestra configurata* NPV-A (MacoNPV-A) but with significant differences. Polypeptide analysis demonstrated similar proteins in occlusion bodies and occlusion derived virions, to those observed in NPVs from *Mamestra* spp. Terminal sequencing revealed that the genome organization shared similarity with that of MacoNPV-A. The most homologous virus was MacoNPV-A 90/2 isolate (95.63% identity and 96.47% similarity), followed by MacoNPV-A 90/4 strain (95.37% and 96.26%), MacoNPV-B (89.21% and 93.53%) and *M*. *brassicae* MNPV (89.42% and 93.74%). Phylogenetic analysis performed with *lef-8*, *lef-9*, *polh* and a concatenated set of genes showed that LaolNPV and the *Mamestra* spp. NPVs clustered together with HaMNPV, but with a closer genetic distance to MacoNPV-A strains. The Kimura 2-parameter (K-2-P) distances of the complete genes were greater than 0.05 between LaolNPV and the MbMNPV/MacoNPV-B/HaMNPV complex, which indicates that LaolNPV is a distinct species. K-2-P distances were in the range 0.015–0.050 for comparisons of LaolNPV with MacoNPV-A strains, such that additional biological characteristics should be evaluated to determine species status. While MacoNPV-A was pathogenic to seven lepidopteran species tested, LaolNPV was only pathogenic to *Chrysodeixis chalcites*. Given these findings, *Lacanobia oleracea nucleopolyhedrovirus* should be considered as a new species in the *Alphabaculovirus* genus.

## Introduction

Baculoviruses have been isolated from more than 700 insect species [1]. The genus *Alphabaculovirus* is the largest genus of baculoviruses, comprising over 90% of the presently known baculoviruses [2–4]. As well, considerable diversity has been observed among different geographical isolates of a single virus species and within baculovirus isolates [5–7], as exemplified by the isolation of multiple genotypes present within a single isolate [6, 8–10].

The alphabaculovirus populations that infect the bertha armyworm, *Mamestra configurata* (Lepidoptera: Noctuidae), represented a good example of this diversity. Populations of this pest are cyclic, with major regional outbreaks occurring every 6–8 years and lasting up to 3 years in western Canada [11, 12]. Epizootics of nucleopolyhedrovirus (NPV) are often associated with collapses of *M*. *configurata* larval populations and these viruses are major mortality factors that may dampen *M*. *configurata* outbreak cycles. A number of NPVs have been isolated from *M*. *configurata* larval populations [13–16]. Notably, two distinct species of alphabaculovirus that are closely related but distinguished by restriction endonuclease profiles, gene content and biological activity have been identified in *M*. *configurata*, namely *Mamestra configurata nucleopolyhedrovirus A* (MacoNPV-A) and *Mamestra configurata nucleopolyhedrovirus B* (MacoNPV-B) [15]. In addition, distinct geographical strains of MacoNPV-A, including 90/2 and 90/4, have been isolated from *M*. *configurata* populations. MacoNPV-A is much more prevalent than MacoNPV-B in western Canada, which might confer the higher infectivity due to host-pathogen evolution [17]. Significantly, MacoNPV-B may be a variant of the European *Mamestra brassicae* NPV (MbMNPV) [3, 4, 6, 11] and appears to have a wider host range than MacoNPV-A [6, 11, 14, 15]. Because of the differences in host range these two viruses could evolve divergently while infecting the same host, resulting in divergence in genomic and phenotypic characteristics [15].

According to the current definition by the International Committee on Taxonomy of Viruses (ICTV), a virus species is a polythetic class of viruses that constitute a replicating lineage and occupy a particular ecological niche [18]. A polythetic class is one whose members have several properties in common, although they do not necessarily all share a single common defining property. In the case of baculoviruses, Jehle et al. [3, 4] proposed the use of genome sequence-based phylogenies in addition to morphological and biological characteristics for classification.

Specifically, they suggested the use of a two-parameter model (K-2-P) proposed by Kimura et al. [19], to estimate the phylogenetic distance between two viruses. Using this method, the viruses in question should be considered as the same species when the K-2-P estimated distance between single genes or concatenated *polh*, *lef-8* and *lef-9* nucleotide sequences is less than 0.015. However, when this distance is larger than 0.05 the baculovirus strains should be considered as constituting different species. For viruses with K-2-P distances between 0.015 and 0.05, complementary information would be necessary for species demarcation. In this sense, morphological, pathological and ecological characteristics such as host species or geographical origin should be taken into account when classifying these viruses [4, 20].

During a sampling program in alfalfa crops near Montpellier in 2011 several lepidopteran larvae, presumably *Lacanobia oleracea* (synonymous of *Mamestra oleracea*), were collected with the typical signs of NPV infection. The present study aimed to characterize these isolates using molecular and phylogenetic tools in combination with host range studies to establish a key aspect of the ecology of the virus. Therefore, the discovery of NPV *L*. *oleracea* infected larvae in France offered an opportunity to apply the established baculovirus species demarcation criteria to determine whether these isolates represent a novel baculovirus species.

## Materials and methods

### Virus isolates, insect source and rearing

In April 2011, lepidopteran larvae were collected from alfalfa crops and the weeds present (mainly grasses and wild brassicas) near Montpellier, France. Two sites were examined for the presence of Lepidoptera; one located beside the village of Lattes (43°33'13.9"N, 3°56'10.0"E) and the other beside the village of Marsillargues (43°39'15.0"N 4°11'25.9"E) in southern France. These fields had been used for many years for the production of pasture and alfalfa. No specific permissions were required for access to the land and the field studies did not involve endangered or protected species.

The noctuids *Agrochola lychnidis*, *Lacanobia oleracea* and *Xylena exsoleta* were identified as the most abundant species present, whereas *Aporophyla australis* occurred in low numbers. In this region *L*. *oleracea* is common, especially during warm summers (M. Frayssenet, pers. obs.), although the larvae and adults of this species can be confused with *A*. *lychnidis*.

A total of 26 larvae were collected and individually reared on semisynthetic diet at 23–25°C. During laboratory rearing seven larvae showed the typical signs of lethal polyhedrosis disease. It was difficult to identify the host species; we believe these insects were *L*. *oleracea*, but we cannot exclude the possibility that one or more of them was *A*. *lychnidis*. Consequently, we will describe the novel isolate as Lacanobia oleracea NPV (LaolNPV) in the following text.

These isolates were compared with previously characterized viruses from our virus collections, namely Chrysodeixis chalcites nucleopolyhedrovirus (ChchSNPV [21]), Helicoverpa armigera nucleopololyhedrovirus (HearNPV [22]), Spodoptera littoralis nucleopolyhedrovirus (SpliNPV [23]), Spodoptera exigua multiple nucleopolyhedrovirus (SeMNPV [24]), Mamestra brassicae multiple nucleopolyhedrovirus (MbMNPV from the Mamestrin bioinsecticide [25]), Mamestra configurata NPV A (MacoNPV-A [14]) and Mamestra configurata NPV B (MacoNPV-B [15]). Once the phylogenetic relationships had been determined, the novel virus isolate was also compared at the biological level with closely related viruses.

The noctuid larvae used in the present study were *Mamestra brassicae*, *Chrysodeixis chalcites*, *Spodoptera littoralis*, *S*. *frugiperda*, *S*. *exigua* and *H*. *armigera*. These were obtained from laboratory colonies reared at the Universidad Pública de Navarra (UPNA) at 25±1°C, 70±5% relative humidity and 16:8 h day:night photoperiod on a semi-synthetic diet [26]. Bioassays involving *Trichoplusia ni* and *Mamestra configurata*, were performed in the Saskatoon Research and Development Centre, Agriculture and Agri-Food Canada at 21±1°C, 60±5% relative humidity and 16:8 h day:night photoperiod using a semi-synthetic diet [27].

### Molecular characterization

#### OB purification, DNA extraction and restriction endonuclease analysis

Occlusion bodies (OBs) of the different isolates were extracted from dead larvae by homogenizing the cadavers in 500 μl of 0.1% (wt/vol) sodium dodecyl sulfate (SDS), filtered through muslin and centrifuged at 2,500 x *g* for 5 min. Pellets were resuspended twice in 500 μl of 0.1% SDS and centrifuged for 5 min at 2,500 x *g*. The resulting pellets were washed twice in distilled water, resuspended in ~200 μl distilled water and OBs were stored at 4°C until required.

For DNA extraction, virions were released from OBs by dissolving the polyhedrin matrix by mixing 100 μl of purified OB suspensions, comprising ~10^8^ OBs, with 100 μl of 0.5 M Na_2_CO_3_ and 50 μl of 10% SDS in a final volume of 500 μl followed by incubation at 60°C for 10 min. Undissolved OBs and other debris were pelleted at 6,000 x *g* for 5 min. The virion-containing supernatant was transferred to sterile 1.5 ml vials and incubated at 50°C with 25 μl proteinase K (20 mg/ml) for 1 h. Viral DNA was extracted twice with phenol (pH 7.8):chloroform (1:1). DNA was precipitated by addition of 10% (v/v) 3 M sodium acetate (pH 5.2) and 2.5 volumes of ice-cold 96% ethanol at 12,000 x *g* for 10 min. The DNA pellet was washed with 70% cold ethanol and centrifuged for 5 min. The ethanol was discarded and DNA was dried at room temperature for 5 min. Finally, DNA pellets were resuspended in 50–100 μl of 0.1X TE buffer (10 mM Tris, 1 mM EDTA), and kept at 4°C until use. For comparison, DNA was also extracted from OBs of ChchNPV, HearNPV, SpliNPV, SeMNPV, MbMNPV, MacoNPV-A and MacoNPV-B.

For restriction endonuclease (REN) digestion, a sample of viral DNA (1–2 μg) was incubated with one of the following enzymes BamHI, BglII, EcoRI or PstI (Takara) (10 U) at 37°C for 4–12 h. Each reaction was stopped by the addition of 4 μl of 6x loading buffer (0.25% w/v bromophenol blue and 40% w/v sucrose). Fragments were separated by electrophoresis using horizontal 1% agarose gels in TAE buffer (0.004 M Tris-acetate, 0.001 M EDTA, pH 8.0) at 16 V for 14 h. DNA fragments were stained with ethidum bromide and visualized on a UV transilluminator (GeneSnap, Syngene). DNA fragment sizes were estimated by comparison to a standard molecular weight marker (Tandem ladder, Lonza, Rockland, USA).

#### Nucleocapsid packaging

To determine whether the virus under study was a single or multiple nucleocapsid NPV, occlusion derived virions (ODVs) were harvested by treating 5x10^8^ OBs with an equal volume of 0.1 M Na_2_CO_3_, for 30 min at 28°C. Undissolved OBs and other debris were pelleted and discarded by low-speed centrifugation (2,500 *x g*, 2 min). The resulting suspensions containing the ODVs were placed on the top of continuous 30–60% (w/w) sucrose gradients and centrifuged at 30,000 *x g* for 1 h at 4°C in a Beckman Ti28 rotor. The banding pattern was visually inspected, photographed and compared with other viruses.

#### Polypeptide analysis

Structural polypeptides from the purified OBs and occlusion derived virions (ODVs) were analyzed on 11% SDS-polyacrylamide slab gels (SDS-PAGE). ODVs were harvested from OBs by mixing 10 μl of purified OBs at 10^10^ OBs/ml with an equal volume of 0.1 M Na_2_CO_3_ and incubating at 28°C for 30 min. Undissolved OBs and other debris were pelleted at 6,000 x *g* for 5 min. A 10 μl volume of purified OBs at a concentration of 10^10^ OBs/ml and 10 μl of harvested ODVs were solubilized with an equal volume of 2x Laemmli sample buffer (65.8 mM Tris-HCl, pH 6.8, 2.1% SDS, 26.3% (w/v) glycerol, 0.01% bromophenol blue, BioRad) by heating to 100°C for 5 min prior to electrophoresis. Electrophoresis was performed at 50 mA during 2 h. Finally, the gels were stained in Comassie Brilliant Blue R solution (0.1% Comassie R Brilliant Blue, 10% v/v acetic acid and 50% v/v ethanol) for 30 min and destained with a bleaching solution (9.45% v/v ethanol and 6.75% v/v acetic acid). The polypeptide profiles were visually inspected and photographed.

#### DNA cloning, sequence analysis and phylogenetic analysis

In order to obtain genomic sequence information four genomic fragments were terminally sequenced. For this, two genomic libraries were constructed in pUC19 (New England Biolabs) using the EcoRI or PstI digested DNAs. Briefly, 2 μg of viral DNA extracted from purified OBs were digested with EcoRI or PstI overnight at 37°C and then heated to 65°C during 15 min to inactivate enzymes. A 5 μg sample of pUC19 vector was digested with EcoRI or PstI in the same conditions. The digested vector (20 μl) was dephosphorylated during 2h at 37°C using an alkaline phosphatase (Roche Life Science, Basel, Switzerland). After dephosphorylation, the DNA (100 μl) was purified in low melting agarose gel using commercial kit for gel extraction (PCR clean-up, gel extraction, Macherey-Nagel, Düren, Germany) following manufacture’s instructions. A 50 ng sample of the purified vector was ligated with 5 μl of the digested viral DNA (100 ng/μl of total fragmented DNA) using T4 DNA ligase (New England Biolabs) at 16°C overnight. After ligation, DH5α cells were transformed with the recombinant plasmids and plated on LB agar containing 100 μg/ml ampicillin, 1 μM IPTG and 80 μg/ml X-Gal. A total of 50 white colonies for each ligation were amplified in LB broth containing 100 μg/ml ampicillin. Plasmid DNAs were purified by alkaline lysis and screened for the presence of EcoRI or PstI inserts by digestion with the respective enzymes followed by electrophoresis in 1% agarose gel. Inserts were authenticated by comparing their migration in agarose gels with the fragments of the viral DNA generated by the digestion with the same enzymes.

Two EcoRI and two PstI fragments (EcoRI-4Kb, EcoRI-6kb, PstI-5kb and PstI-6kb) were selected for terminal sequencing. Terminal nucleotide sequences were determined by Sanger Sequencing method performed by Stab Vida Company (Caparica, Portugal), employing standard M13 forward and M13 reverse primers. Sequence information was analyzed for the presence of open reading frames (ORFs) and for domain prediction using Clone Manager 9.0 (Scientific and Educational Software Server). Homology searches were performed both at the nucleotide and deduced amino acid levels, for all putative ORFs. DNA and protein comparisons with entries in the updated GenBank/EMBL, SWISS-PROT and PIR databases were performed using BLATn, BLASTp and FASTA programs [28, 29].

For phylogenetic analyses, the DNA sequences within the coding regions of three highly conserved genes, the *late expression factor 8* (*lef-8*), *late expression factor 9* (*lef-9*) and *polyhedrin* (*polh*), were used [4, 30, 31]. As recommended by Jehle et al. [4], for viruses with Kimura two-parameter (K-2-P) distances of more than 0.015 in the marker genes, the complete sequences of these three marker genes were determined and used in phylogenetic analyses. For this, primers were designed in the *polh*, *lef-8* and *lef-9* genes based on the genomic sequence of the most homologous NPV. The primer pairs used were MacoA-polh-F (ATGTATACCCGTTATAGTTA)-MacoA-polh-R (TTAGTAAGCCGGTCCGTTGTA), MacoA-lef8-F (ATGACGGACGTGATTGACGA)-MacoA-lef8-R (TCATCGAACCACTGTGTTGTG), and MacoA-lef9-F (ATGACCTTTAGCGGTCATTC)-MacoA-lef9-R (CTAGTCCAAAAACATGTCGA). The resulting fragments were cloned into pGEM-T Easy vector (Promega) following manufacturer’s instructions and transformed as previously described. Two clones for each gene were selected and nucleotide sequences were determined by Sanger Sequencing method performed by Stab Vida Company (Caparica, Portugal), employing standard SP6 and T7 reverse primers.

The K-2-P distances were calculated for single and concatenated *lef-8*, *lef-9* and *polh* genes. Concatenated sequences from the same virus were treated as a single sequence. Multiple sequence alignments were performed using MEGA6.06 software [32], and the K-2-P nucleotide substitution model was used for the analysis. Maximum parsimony (MP) phylogenetic trees (1,000 bootstrap replicates) were inferred from the nucleotide sequence alignments using MEGA 6.06. Introduced gaps were treated as missing data.

Genome sequences used in the comparative analysis were obtained from GenBank (accession number included as well as the publication): Autographa californica (Ac) MNPV (NC_001623/L22858 [33]), Agrotis ipsilon (Agip) NPV (NC_011345 [34]), Agrotis segetum (Agse) NPV (NC_007921 [35]), Bombyx mori (Bm) NPV (NC_001962 [36]), Chrysodeixis chalcites (Chch) NPV (NC_007151 [37]), Helicoverpa armigera multiple (HearM) NPV (NC_011615 [38]), Helicoverpa armigera single (HearS) NPV (AF303045 [39]), Helicoverpa zea (Hz) NPV (AF334030 [40]), Lymantria dispar (Ld) MNPV (AF081810 [41]), Mamestra brassicae (Mb) MNPV (NC_023681/JQ798165 [42]), Mamestra configurata A 90/2 (MacoA 90/2) NPV (U59461/AF467808 [14]), Mamestra configurata A 90/4 (MacoA 90/4) NPV (AF539999 [16]), Mamestra configurata B (MacoB) NPV (AY126275 [15]), Rachiplusia ou (Ro) MNPV (AY145471 [43]), Spodoptera exigua (Se) MNPV (AF169823 [44]), Spodoptera frugiperda (Sf) MNPV (HM595733 [45]), Spodoptera litura (Splt) NPV (NC_003102/AF325155 [46]) and Trichoplusia ni (Tn) NPV (NC_007383/DQ017380 [47]).

### Biological characterization

#### Host range determination

The host range of LaolNPV was determined by oral inoculation bioassays with the following lepidopteran species: *M*. *brassicae*, *M*. *configurata*, *T*. *ni*, *C*. *chalcites*, *S*. *littoralis*, *S*. *frugiperda*, *S*. *exigua* and *H*. *armigera*. The results of the oral inoculation studies were compared with those of MbMNPV, MacoNPV-A and MacoNPV-B. For this, second-instar larvae from the laboratory colonies were starved for 8–12 h at 26°C and then allowed to drink from an aqueous suspension containing 10% sucrose (w/v) and Fluorella blue and OBs at concentrations of 10^5^ and 10^7^ OBs/ml, following the droplet feeding method [48]. These concentrations were selected to permit determination of the susceptible host species based on preliminary tests (data not shown) and previous studies using MacoNPV [13, 15, 16] or MbMNPV [49, 50]. Larvae that ingested the suspension within 10 min, which were identified by the blue color of their intestine, were transferred to individual wells of a 25-well tissue culture plate with a cube (1 cm^3^) of semisynthetic diet [26]. Host range tests were performed using 25 larvae per virus concentration and 25 larvae treated with aqueous suspension containing sucrose and Fluorella blue without OBs as controls. Each assay comprised three replicates. Larvae were reared individually at 25°C and mortality was recorded every 24 h until the insects had either died or pupated. Dead larvae were examined microscopically to determine the presence of OBs. When present, OBs were purified from each group of dead larvae and viral DNA was extracted as previously described and subjected to REN analysis to determine the identity of the virus. The percentage of larval mortality was calculated for each virus and concentration and subjected to univariante analysis of variance (ANOVA) in SPSS v23 (IBM SPSS Statistics, Softtonic). The significance of treatments was determined for comparisons among the estimated means by Tukey test (p<0.05).

## Results

### Molecular characterization

#### REN profiles indicated the proximity of LaolNPV to MacoNPV-A

**Fig 1:**
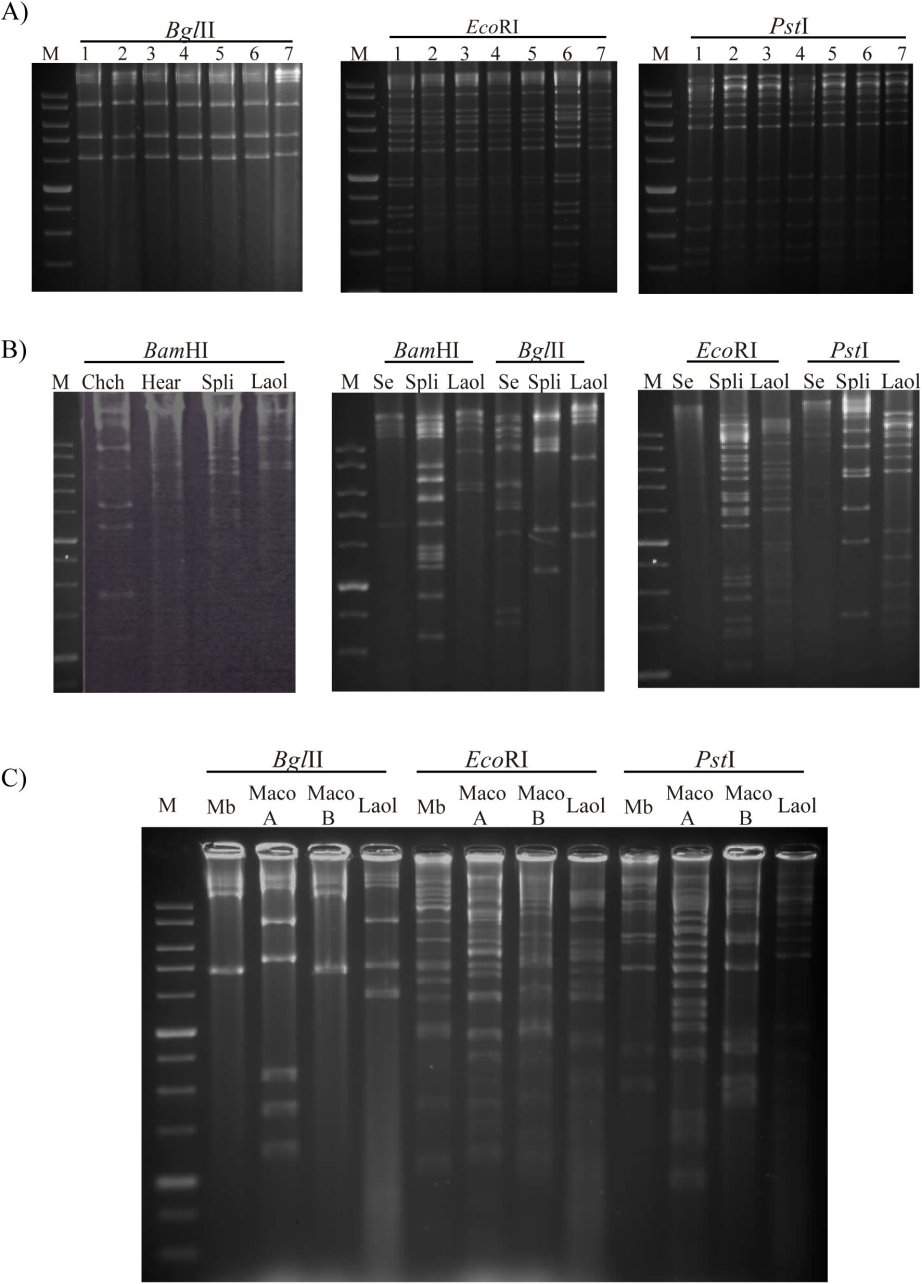
Restriction endonuclease analysis of LaolNPV DNA. Restriction endonuclease profiles following digestion of A) genomic DNAs of the seven isolates of LaolNPV collected in alfalfa crops nearby Montpellier with BglII, EcoRI and PstI enzymes, B) genomic DNAs of Chrysodeixis chalcites NPV (Chch), Helicoverpa armigera SNPV (Hear), Spodoptera littoralis (Spli), Spodopera exigua MNPV (Se) and the virus under study (LaolNPV) with BamHI, BglII, EcoRI and PstI, and C) genomic DNAs of Mamestra brassicae MNPV (Mb), Mamestra configurata NPV-A (MacoA), Mamestra configurata NPV-B (MacoB) and LaolNPV with BglII, EcoRI and PstI.

The BglII, EcoRI and PstI profiles of the isolates from each of the seven insects showed very similar patterns with the different enzymes tested. No submolar bands were observed in the different REN profiles, indicating that each isolate comprised a single or majority genotype (Fig 1A). Therefore, the first isolate was selected as the prototype and its DNA and OBs were used for subsequent studies.

When comparing the REN profiles with those of ChchNPV, HearNPV, SeMNPV or SpliNPV, marked differences were observed, suggesting that the novel virus was genetically distinct from the other virus species tested (Fig 1B). Additionally comparison with the REN profiles generated *in silico* with AcMNPV, LdMNPV or TnSNPV also indicated that LaolNPV was genetically distinct from these viruses (data not shown).

The REN profiles were then compared with those of MbMNPV, MacoNPV-A and MacoNPV-B. As observed in the BglII profiles (Fig 1C) the LaolNPV isolate from France might be more similar to MacoNPV-A than to MbMNPV or MacoNPV-B, although clear differences among these viruses were observed in the EcoRI and PstI profiles, suggesting that LaolNPV might represent a novel virus species.

#### LaolNPV is a multiple nucleocapsid NPV

**Fig 2:**
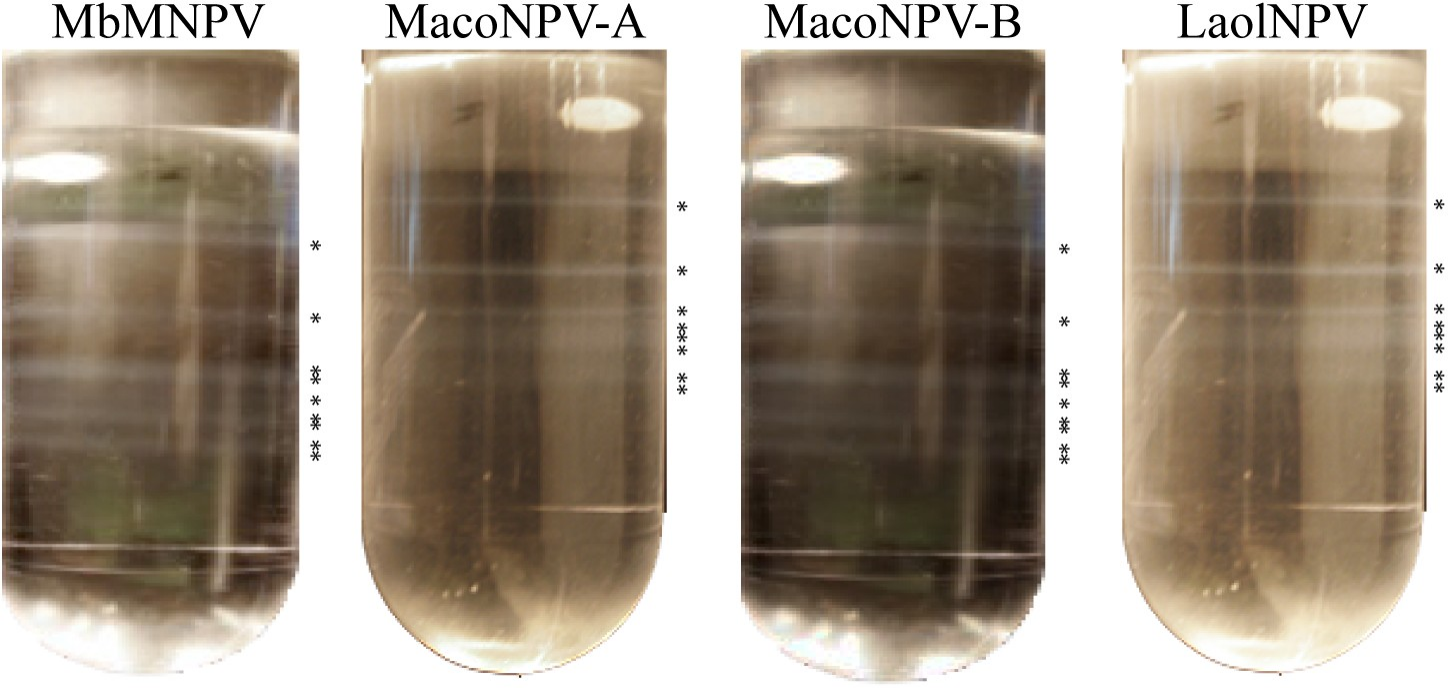
ODV banding patterns. ODV banding patterns of MbMNPV, MacoNPV-A, MacoNPV-B and LaolNPV after continous sucrose grandient separation. Asterisks indicate the position of the different visible bands.

The banding pattern observed following ODV density gradient centrifugation revealed that the ODVs of LaolNPV contained multiple nucleocapsids, as indicated by the multiple bands visible in the sucrose gradient (Fig 2). Based on visual inspection, the banding patterns of MbMNPV, MacoNPV-A and MacoNPV-B were quite similar, and comprised between 1 and 9 nucleocapsids/ODV in MbMNPV and MacoNPV-B compared to between 1 and 8 nucleocapsids/ODV in MacoNPV-A and LaolNPV.

#### OB and ODV structural polypetides in LaolNPV

**Fig 3:**
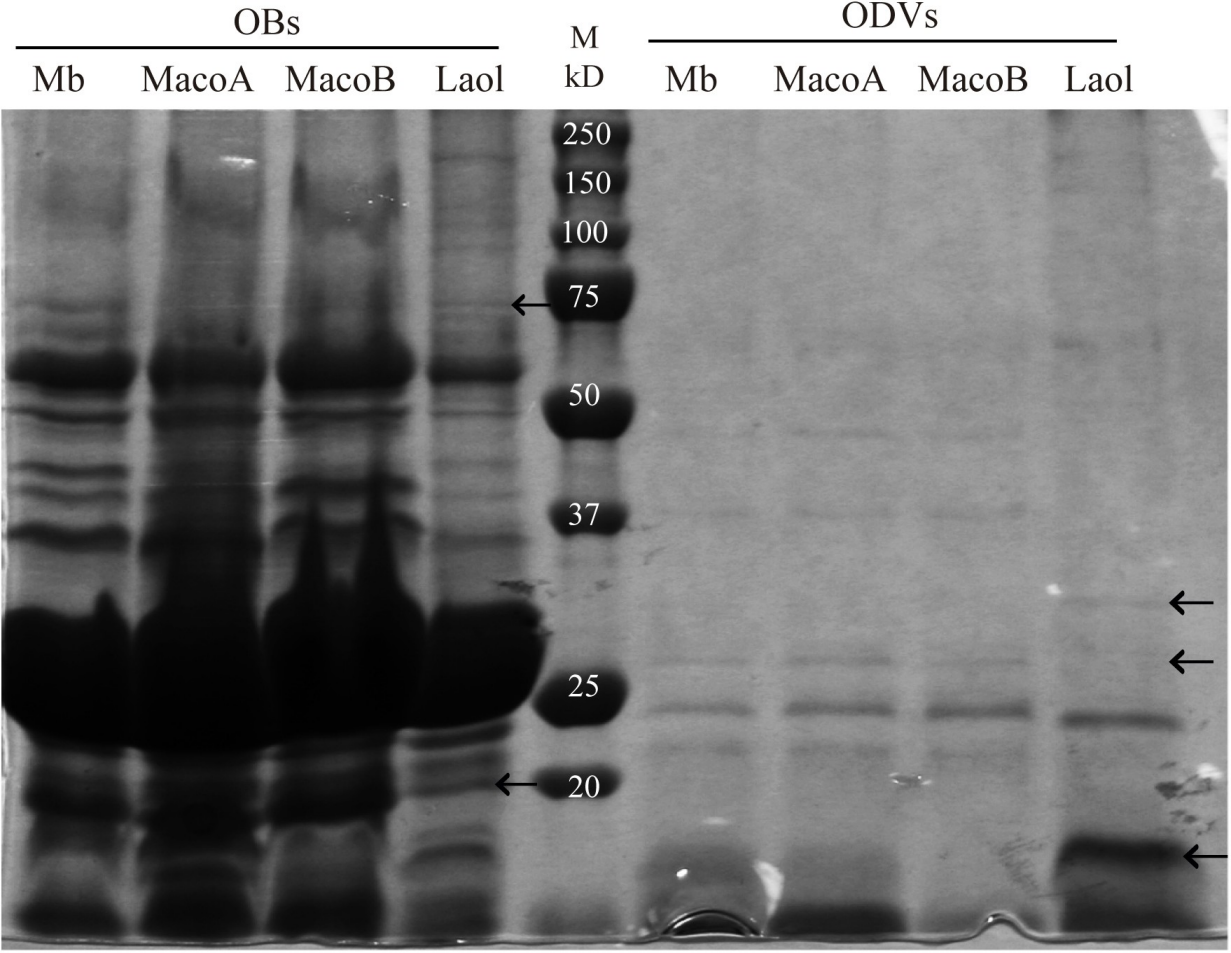
OB and ODV structural polypeptides patterns. OB and ODV structural polypeptides profiles of MbMNPV (Mb), MacoNPV-A (MacoA), MacoNPV-B (MacoB) and the virus under study (LaolNPV). OBs and harvested ODVs after solubilization were electrophoresed on an 11% SDS-PAGE gel. The molecular weights of the fragmets of the Precision Plus Protein All Blue marker, from Bio-Rad, (M) are indicated above each band. The arrows indicated the differences observed in the presence and absence of proteins fragments and in differences in molecular weights.

The SDS-PAGE profiles of OB and ODV polypeptides were generally similar between LaolNPV and the other three NPVs (Fig 3). There were, however, clear differences in the molecular weights of specific major proteins and the presence or absence of particular proteins. In the OB polypeptide profiles, the virus under study presented a band at 75 kDa that was absent in MbMNPV, MacoNPV-A and MacoNPV-B. Additionally, the intensity of the band at 20 kDa was much reduced in LaolNPV compared to that of the other three viruses. Similarly, in the ODV profiles, LaolNPV presented a clear band of ~30 kDa that was absent in the other three viruses, while the band below the 25 kDa marker showed a lower molecular weight and the band of ~10 kDa had a stronger intensity in LaolNPV than in the other three viruses.

#### Terminal sequencing and phylogenetic analysis indicates that LaolNPV evolved from the same ancestor as MacoNPV-A

The terminal sequence information obtained from the four cloned fragments showed homology with 18 ORFs. However, although they appeared distinct in agarose gels, the two PstI fragments that were terminal sequenced were in fact identical fragments (corresponding to the 5,575 bp [nt 6,124 to 11,699] PstI fragment in the MacoNPV-A 90/2 genome [14]), showing the same ORFs. Therefore, just a total of 14 different ORFs were identified as being homologues of known baculovirus ORFs and all were assigned on the basis of their similarity to previously identified ORFs in the MacoNPV-A 90/2 genome [14]. The direction of transcription, its position in the sequenced fragment relative to that in the MacoNPV-A 90/2 genome, as well as its identity to MacoNPV-A 90/2 homologs are summarized in Table 1. Within the 5' end of PstI-5kb (M13-Rv sequence) two ORFs were identified, namely *orf5* and *odpv-6e* that showed 93% and 95% nt sequence identity with the homologous ORFs of MacoNPV-A 90/2, while the 3’ end (M13-Fw sequence) included *efp* and *orf10*, with 97 and 99% nt identity to the corresponding homologs in MacoNPV-A 90/2, respectively (Table 1). Similarly, within the PstI-6kb 5’-terminal sequence (M13-Fw) *orf5* and *odpv-6e* were identified, showing 93 and 96% identity to homologous ORFs in the MacoNPV-A 90/2 genome, respectively, while the 3’-terminal sequence (M13-Rv) included *efp* and *orf10* ORFs, showing 95 and 99% identity. EcoRI-4kb is the 3,899 bp fragment with homology to the MacoNPV-A 90/2 genome region located between nt 41,533 to 45,533. The M13-Fw sequence included *orf44*, *pkip-1* and *orf46* with 94, 95 and 97% of identity, respectively. The M13-Rv sequence included *pif2* and *pif1*, both with identities of 96%. Finally, the EcoRI-6kb is the 5,818 bp fragment homologous to nt 97,657 to 97,474 in the MacoNPV-A 90/2 genome. Within the M13-Fw sequence the *cg30* and *vp91* genes were located with identities of 95 and 98%, respectively. The M13-Rv sequence contained the *gp41*, *orf105* and *vlf-1* genes with identities of 98, 97 and 93%, to MacoNPV-A 90/2 homologues, respectively (Table 1).

**Table 1:** ORFs identified in the terminal sequences of four restriction fragments. The position and orientation of the 14 putative ORFs in the most homologous baculovirus MacoNPV-A 90/2 genome [14] along with the identity at nucleotide level and the percentage of gaps are also indicated.

ORF num	Gene family	Size (nt)	Genomic fragment	Primer	Sequence size (nt)	Position in the fragment (nt)	Most homologous ORF	Position in MacoNPV-A 90/2	MacoNPV-A 90/2		MacoNPV-A 90/4	
									Identities	Gaps	Identities	Gaps
1	*orf5*	176	PstI-5kb	M13-Rv	1,187	9>185	Maco5	6,124>6,312	175/189 (93%)	12/189 (6%)	175/189 (93%)	12/189 (6%)
2	*odpv-6e*	864	PstI-5kb	M13-Rv	1,187	266>1,129	Maco6	6,400>7,263	825/864 (95%)	0/864 (0%)	822/864 (95%)	3/864 (0%)
3	*efp*	845	PstI-5kb	M13-Fw	1,179	30>874	Maco9	10,566>11,410	819/845 (97%)	0/845 (0%)	821/845 (97%)	0/845 (0%)
4	*orf10*	189	PstI-5kb	M13-Fw	1,179	972<1,160	Maco10	11,516<11,704	187/189 (99%)	0/189 (0%)	187/189 (99%)	0/189 (0%)
1	*orf5*	177	PstI-6kb	M13-Fw	1,085	17>193	Maco5	6,124>6,312	175/189 (93%)	12/189 (6%)	175/189 (93%)	12/189 (6%)
2	*odpv-6e*	812	PstI-6kb	M13-Fw	1,085	274>1085	Maco6	6,400>7,211	777/812 (96%)	0/812 (0%)	774/812 (95%)	3/812 (0%)
3	*efp*	458	PstI-6kb	M13-Rv	1,051	1>458	Maco9	10,953>11,410	437/458 (95%)	0/458 (0%)	437/458 (95%)	0/458 (0%)
4	*orf10*	190	PstI-6kb	M13-Rv	1,051	556<745	Maco10	11,516<11,705	188/190 (99%)	0/190 (0%)	188/190 (99%)	0/190 (0%)
5	*orf44*	345	EcoRI-4kb	M13-Fw	1,177	21>365	Maco44	41,562>41,894	323/345 (94%)	12/345 (3%)	323/345 (94%)	12/345 (3%)
6	*pkip-1*	510	EcoRI-4kb	M13-Fw	1,177	388>897	Maco45	41,917>42,427	488/511 (95%)	1/511 (0%)	488/510 (96%)	0/510 (0%)
7	*orf46*	259	EcoRI-4kb	M13-Fw	1,177	919<1,177	Maco46	42,449<42,707	252/259 (97%)	0/259 (0%)	252/259 (97%)	0/259 (0%)
8	*pif2*	144	EcoRI-4kb	M13-Rv	1,011	3>146	Maco48	44,540>44,683	138/144 (96%)	0/144 (0%)	138/144 (96%)	0/144 (0%)
9	*pif1*	739	EcoRI-4kb	M13-Rv	1,011	161>899	Maco49	44,698>45,436	707/739 (96%)	0/739 (0%)	708/739 (96%)	0/739 (0%)
10	*cg30*	611	EcoRI-6kb	M13-Fw	1,206	19>617	Maco100	91,689>92,299	582/611 (95%)	12/611 (1%)	578/611 (95%)	12/611 (1%)
11	*vp91*	450	EcoRI-6kb	M13-Fw	1,206	679<1,128	Maco101	92,662<92,811	441/450 (98%)	0/450 (0%)	435/444 (98%)	0/444 (0%)
12	*gp41*	510	EcoRI-6kb	M13-Rv	1,203	2>511	Maco104	96,354>96,863	501/510 (98%)	0/510 (0%)	501/510 (98%)	0/510 (0%)
13	*orf105*	345	EcoRI-6kb	M13-Rv	1,203	508>852	Maco105	96,860>97,063	337/346 (97%)	1/346 (0%)	337/345 (98%)	0/345 (0%)
14	*vlf-1*	273	EcoRI-6kb	M13-Rv	1,203	854–1126	Maco106	97,207>97,479	255/273 (93%)	2/273 (0%)	270/273 (99%)	0/273 (0%)
Mean									95.89%	0.88%	96.28%	0.88%

The amino acid sequence identity and similarity of the 14 ORFs were also compared with those of other NPVs (Table 2). The most homologous NPV to LaolNPV was MacoNPV-A 90/2 with 95.93% identity and 96.64% similarity at the amino acid level, followed by MacoNPV-A 90/4 with 95.78% identity and 96.50% similarity. The identity and similarity values to MbMNPV (90.00 and 93.79%) and MacoNPV-B (89.78 and 93.64%), were lower, but were of a similar magnitude to the identity and similarity values (90.42 and 95.00%) for comparisons of MacoNPV-A 90/2 and MacoNPV-B or MbMNPV (data not shown). In contrast, the identity and similarity values for comparisons of the MacoNPV-A 90/2 and MacoNPV-A 90/4 isolates, using the same sequences, increased to 99.53 and 99.74%, respectively (data not shown). Similar results were obtained at the nucleotide level: the nt sequence identity between LaolNPV and the MacoNPV-A isolates was intermediate (95.89%) between that found between the same species (isolates of MacoNPV-A; 99.68%), and different species (MacoNPV-A 90/2 and MacoNPV-B or MbMNPV; 90.42 and 88.95%, respectively).

**Table 2:** Percentages of LaolNPV amino acid sequence identity to homologous ORFs in other baculoviruses.

Protein family	Size (aa)	Protein position (aa)										
		Identity (%)										
		Positive (%)										
		Gaps (%)										
		MacoNPV-A (90/2)	MacoNPV-A (90/4)	MacoNPV-B	MbMNPV	HearMNPV	AgseNPV	SfMNPV	ChchNPV	TnSNPV	LdMNPV	AcMNPV	
ORF5	58	141 to 202 aa	141 to 202 aa	129 to 190 aa	126 to 187 aa	126 to 187 aa	-	-	-	-	-	-
		56/62 (90%)	56/62 (90%)	45/62 (73%)	45/62 (73%)	45/62 (73%)	-	-	-	-	-	-
		56/62 (90%)	56/62 (90%)	52/62 (83%)	53/62 (85%)	53/62 (85%)	-	-	-	-	-	-
		4/62 (6%)	4/62 (6%)	4/62 (6%)	4/62 (6%)	4/62 (6%)	-	-	-	-	-	-
ODVP-6E	288	1 to 288	1 to 287 aa	1 to 288 aa	1 to 288 aa	1 to 288 aa	3 to 287 aa	2 to 286 aa	3 to 287 aa	3 to 287 aa	2 to 287 aa	2 to 287 aa
		275/288 (95%)	269/288 (93%)	256/288 (89%)	256/288 (89%)	256/288 (89%)	169/287 (59%)	153/287 (53%)	165/287 (57%)	165/287 (57%)	150/289 (52%)	147/289 (51%)
		281/288 (97%)	276/288 (95%)	271/288 (94%)	271/288 (94%)	271/288 (94%)	204/287 (71%)	163/266 (61%)	205/287 (71%)	205/287 (71%)	201/289 (69%)	189/289 (65%)
		0/288 (0%)	1/288 (0%)	0/288 (0%)	0/288 (0%)	0/288 (0%)	2/287 (0%)	11/266 (4%)	4/287 (1%)	4/287 (1%)	6/289 (2%)	6/289 (2%)
EFP	280	401 to 680 aa	401 to 680 aa	398 to 678 aa	398 to 678 aa	398 to 678 aa	403 to 679 aa	403 to 666 aa	390 to 616 aa	390 to 615 aa	390 to 615 aa	405 to 493 aa
		273/280 (98%)	273/280 (98%)	259/281 (92%)	260/281 (93%)	260/281 (93%)	116/280 (41%)	108/266 (41%)	91/232 (39%)	89/231 (39%)	95/286 (33%)	29/96 (30%)
		277/280 (98%)	277/280 (98%)	272/281 (96%)	272/281 (96%)	272/281 (96%)	169/280 (60%)	163/266 (61%)	147/232 (63%)	146/231 (63%)	140/286 (48%)	46/96 (47%)
		0/280 (0%)	0/280 (0%)	1/281 (0%)	1/281 (0%)	1/281 (0%)	9/280 (3%)	11/266 (4%)	9/232 (3%)	8/231 (3%)	40/286 (13%)	10/96 (10%)
ORF10	62	258 to 319 aa	258 to 319 aa	258 to 319 aa	258 to 319 aa	258 to 319 aa	275 to 335 aa	-	-	-	-	-
		62/62 (100%)	62/62 (100%)	59/62 (95%)	59/62 (95%)	60/62 (97%)	40/61 (66%)	-	-	-	-	-
		62/62 (100%)	62/62 (100%)	61/62 (98%)	61/62 (98%)	61/62 (98%)	47/61 (77%)	-	-	-	-	-
		0/62 (0%)	0/62 (0%)	0/62 (0%)	0/62 (0%)	0/62 (0%)	0/61 (0%)	-	-	-	-	-
ORF44	114	64 to 173 aa	123 to 232 aa	64 to 175 aa	64 to 175 aa	64 to 175 aa	79 to 185 aa	82 to 181 aa	-	-	-	-
		105/114 (92%)	105/114 (92%)	89/115 (77%)	90/115 (78%)	88/115 (77%)	43/107 (40%)	41/100 (41%)	-	-	-	-
		108/114 (94%)	108/114 (94%)	97/115 (84%)	97/115 (84%)	96/115 (83%)	63/107 (58%)	53/100 (53%)	-	-	-	-
		4/114 (3%)	4/114 (3%)	4/115 (3%)	4/115 (3%)	4/115 (3%)	16/107 (14%)	24/100 (24%)	-	-	-	-
PKIP-1	169	1 to 169 aa	1 to 169 aa	1 to 169 aa	1 to 169 aa	1 to 169 aa	5 to 164 aa	13 to 174 aa	1 to 167 aa	14 to 167 aa	5 to 169 aa	-
		154/164 (94%)	159/169 (94%)	151/169 (89%)	151/169 (89%)	151/169 (89%)	92/160 (58%)	83/162 (51%)	71/168 (42%)	67/159 (42%)	60/170 (35%)	-
		161/164 (98%)	166/169 (98%)	163/169 (96%)	163/169 (96%)	163/169 (96%)	121/160 (75%)	117/162 (72%)	106/168 (63%)	102/159 (64%)	99/170 (58%)	-
		0/164 (0%)	0/169 (0%)	0/169 (0%)	0/169 (0%)	0/169 (0%)	2/160 (1%)	3/162 (1%)	5/168 (2%)	6/159 (3%)	16/170 (9%)	-
ORF46		29 to 113 aa	29 to 113 aa	29 to 113 aa	29 to 113 aa	29 to 113 aa	29 to 112 aa	-	-	-	-	-
		81/85 (95%)	81/85 (95%)	78/85 (92%)	78/85 (92%)	78/85 (92%)	47/84 (56%)	-	-	-	-	-
		81/85 (95%)	81/85 (95%)	81/85 (95%)	81/85 (95%)	81/85 (95%)	56/84 (66%)	-	-	-	-	-
		0/85 (0%)	0/85 (0%)	0/85 (0%)	0/85 (0%)	0/85 (0%)	1/84 (1%)	-	-	-	-	-
PIF-2	47	373 to 419 aa	373 to 419 aa	373 to 419 aa	373 to 419 aa	373 to 419 aa	354 to 398 aa	352 to 396 aa	337 to 381 aa	-	336 to 378 aa	155 to 198 aa
		44/47 (94%)	44/47 (94%)	44/47 (94%)	44/47 (94%)	44/47 (94%)	28/45 (62%)	29/45 (64%)	18/45 (40%)	-	20/43 (47%)	22/44 (50%)
		44/47 (93%)	44/47 (93%)	45/47 (95%)	45/47 (95%)	45/47 (95%)	31/45 (68%)	33/45 (73%)	24/45 (53%)	-	23/43 (53%)	26/44 (59%)
		0/47 (0%)	0/47 (0%)	0/47 (0%)	0/47 (0%)	0/47 (0%)	0/45 (0%)	0/45 (0%)	0/45 (0%)	-	0/43 (0%)	0/44 (0%)
PIF-1	246	1 to 246 aa	1 to 246 aa	1 to 246 aa	1 to 246 aa	1 to 246 aa	1 to 244 aa	18 to 239 aa	19 to 237 aa	19 to 239 aa	13 to 239 aa	17 to 240 aa
		240/246 (98%)	240/246 (98%)	232/246 (94%)	233/246 (95%)	233/246 (95%)	169/245 (69%)	157/223 (70%)	137/226 (61%)	137/228 (60%)	134/232 (58%)	131/225 (58%)
		243/246 (98%)	243/246 (98%)	242/246 (98%)	242/246 (98%)	242/246 (98%)	200/245 (81%)	189/223 (84%)	174/226 (76%)	173/228 (75%)	167/232 (71%)	167/225 (74%)
		0/246 (0%)	0/246 (0%)	0/246 (0%)	0/246 (0%)	0/246 (0%)	1/245 (0%)	1/223 (0%)	7/226 (3%)	7/228 (3%)	6/232 (2%)	1/225 (0%)
CG30	198	78 to 279 aa	78 to 279 aa	78 to 274	78 to 274 aa	78 to 274 aa	-	-	-	-	-	-
		187/202 (93%)	187/202 (93%)	156/206 (76%)	159/206 (77%)	157/206 (76%)	-	-	-	-	-	-
		194/202 (96%)	195/202 (96%)	171/206 (83%)	173/206 (83%)	171/206 (83%)	-	-	-	-	-	-
		4/202 (96%)	4/202 (96%)	17/206 (8%)	17/206 (8%)	17/206 (8%)	-	-	-	-	-	-
VP91	149	661 to 809 aa	662 to 810 aa	664 to 812 aa	664 to 812 aa	664 to 812 aa	630 to 778 aa	672 to 813 aa	645 to 789 aa	675 to 819 aa	702 to 849 aa	428 to 571 aa
		146/149 (98%)	146/149 (98%)	147/149 (99%)	147/149 (99%)	146/149 (98%)	94/150 (63%)	92/143 (64%)	75/145 (52%)	78/1457 (53%)	72/149 (48%)	72/145 (50%)
		148/149 (99%)	148/149 (99%)	149/149 (100%)	149/149 (100%)	148/149 (99%)	116/150 (77%)	121/143 (84%)	106/145 (73%)	104/147 (70%)	99/149 (66%)	106/145 (73%)
		0/149 (0%)	0/149 (0%)	0/149 (0%)	0/149 (0%)	0/149 (0%)	3/150 (2%)	1/143 (0%)	3/145 (2%)	6/147 (4%)	4/149 (2%)	4/145 (2%)
GP41	169	165 to 333 aa	165 to 333 aa	165 to 333 aa	165 to 333 aa	165 to 333 aa	174 to 332 aa	171 to 329 aa	140 to 297 aa	164 to 321 aa	158 to 318 aa	232 to 387 aa
		169/169 (100%)	169/169 (100%)	167/169 (99%)	167/169 (9%)	167/169 (9%)	130/159 (82%)	130/159 (82%)	97/161 (60%)	98/161 (61%)	93/165 (56%)	92/160 (58%)
		169/169 (100%)	169/169 (100%)	167/169 (98%)	167/169 (98%)	167/169 (98%)	147/159 (92%)	147/159 (92%)	120/161 (74%)	122/161 (75%)	118/165 (71%)	118/160 (73%)
		0/169 (0%)	0/169 (0%)	0/169 (0%)	0/169 (0%)	0/169 (0%)	0/159 (0%)	0/159 (0%)	5/161 (3%)	5/161 (3%)	9/165 (5%)	7/160 (4%)
ORF105	114	1 to 65 aa	1 to 114 aa	40 to 151 aa	40 to 151 aa	40 to 151 aa	1 to 124 aa	1 to 108 aa	1 to 116 aa	1 to 119 aa	9 to 102 a	1 to 92 aa
		64/65 (98%)	112/114 (98%)	105/114 (92%)	105/114 (92%)	105/114 (92%)	63/124 (51%)	61/109 (56%)	51/118 (43%)	50/121 (41%)	49/104 (47%)	39/104 (38%)
		64/65 (98%)	112/114 (98%)	110/114 (96%)	110/114 (96%)	110/114 (96%)	79/124 (63%)	72/109 (66%)	79/118 (66%)	72/121 (61%)	68/104 (65%)	57/104 (54%)
		0/65 (0%)	0/114 (0%)	2/114 (1%)	2/114 (1%)	2/114 (1%)	11/124 (8%)	3/109 (2%)	11/118 (9%)	14/121 (11%)	11/104 (10%)	12/104 (11%)
VLF-1	91	1 to 91	1 to 91	1 to 91 aa	1 to 91 aa	1 to 91 aa	1 to 91 aa	1 to 91 aa	2 to 90 aa	2 to 90 aa	21 to 100 aa	5 to 88 aa
		89/91 (98%)	89/91 (98%)	87/91 (96%)	87/91 (96%)	87/91 (96%)	85/94 (90%)	85/91 (93%)	58/89 (65%)	57/89 (64%)	52/80 (65%)	55/84 (65%)
		89/91 (97%)	89/91 (97%)	87/91 (95%)	87/91 (95%)	87/91 (95%)	86/94 (91%)	87/91 (95%)	71/89 (79%)	72/89 (80%)	68/80 (85%)	68/84 (80%)
		0/91 (0%)	0/91 (0%)	0/91 (0%)	0/91 (0%)	0/91 (0%)	0/94 (0%)	0/91 (0%)	1/89 (1%)	1/89 (1%)	1/80 (1%)	1/82 (1%)
Mean Identity	95.93%	95.78%	89.78%	90.00%	90.07%	61.42%	61.50%	51.00%	52.12%	49.00%	50.00%
Mean Similarity	96.64%	96.50%	93.64%	93.79%	93.64%	73.25%	75.10%	68.78%	69.88%	65.11%	65.62%

To further determine the relationship between LaolNPV and the other NPVs and to establish whether this is a novel virus or a variant of MacoNPV-A, a phylogenetic study was performed using a total of 19 NPV sequences and the complete nucleotide sequences of single or concatenated *lef-8*, *lef-9* and *polh* using maximum parsimony (MP) and MEGA6.06 distance methods [4]. The K-2-P model was used to calculate the genetic distances between the different viruses.

**Fig 4:**
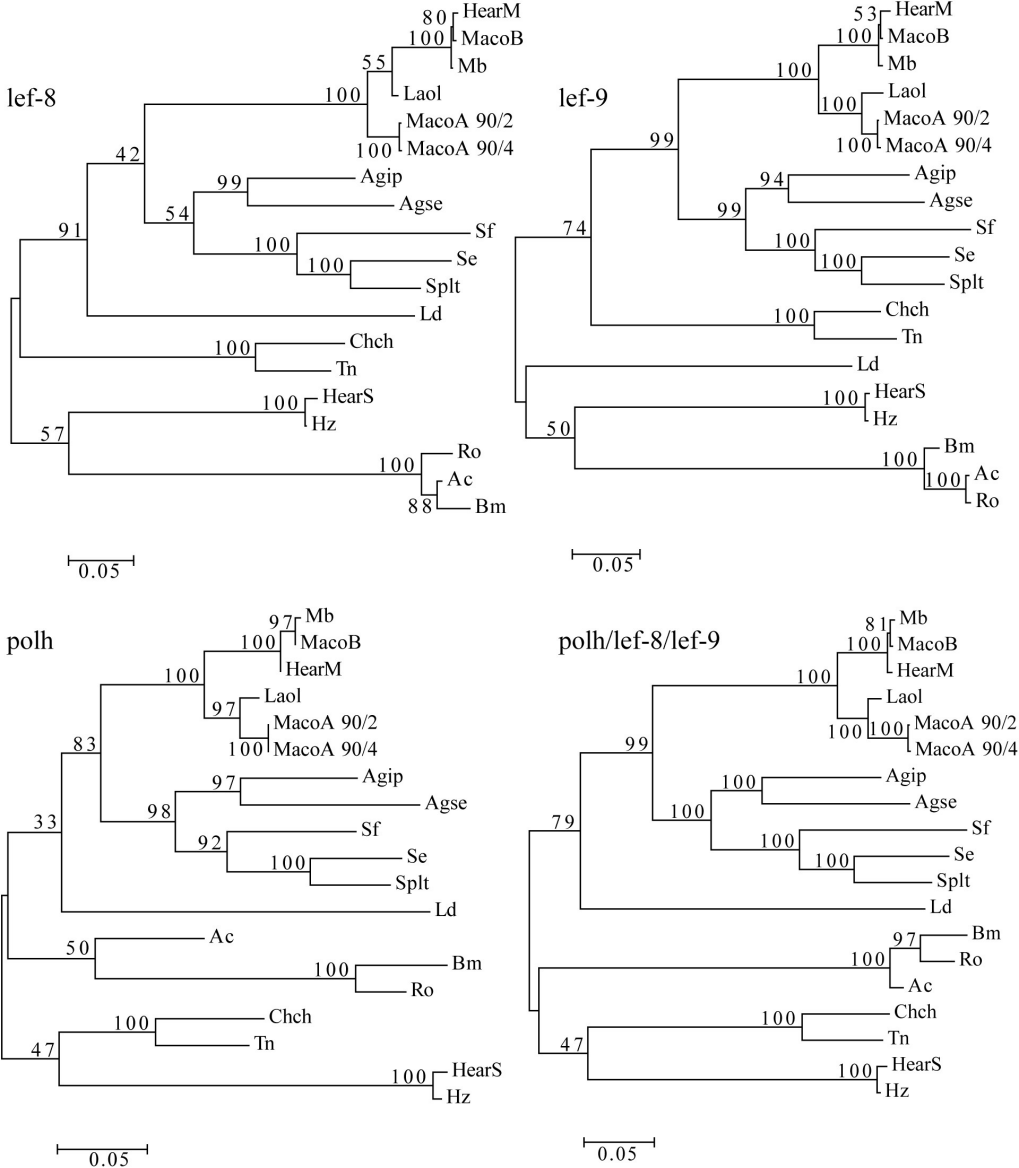
Phylogenetic analysis. Molecular phylogenetic analysis by maximum likelihood method obtained with the complete gene sequences of single or concatenated *lef-8*, *lef-9*, and *polh* genes. The phylogeny was inferred using the maximum likelihood method based on the Kimura two-parameter model. The tree with the highest log likelihood value (-41803.7062) is shown. The percentage of trees in which the associated taxa clustered together is shown next to the branches. Initial tree(s) for the heuristic search were obtained automatically by applying Neighbor-Join and BioNJ algorithms to a matrix of pairwise distances estimated using the Maximum Composite Likelihood (MCL) approach, and then selecting the topology with the superior log likelihood value. The tree is drawn to scale, with branch lengths measured in the number of substitutions per site (shown as values next to the branches). The analysis involved 19 nucleotide sequences. All positions containing gaps and missing data were eliminated. There were a total of 4,702 positions in the final dataset. Analyses were conducted in MEGA6.

The phylogenetic trees using complete genes sequences showed that in the four cases *Mamestra* NPVs formed a separate branch together with HearMNPV and LaolNPV, although with different bootstrap values between branches depending on the gene (Fig 4). MbMNPV shares close sequence identity with MacoNPV-B and HearMNPV, whereas MacoNPV-A strains formed a separate branch. In all cases, except the *lef-8* gene, LaolNPV clustered on the same branch as MacoNPV-A isolates, clearly separated from the MbMNPV/MacoNPV-B/HaMNPV complex. MacoNPV-A 90/2 and MacoNPV-A 90/4 invariably clustered together, whereas LaolNPV, although clearly proximal, was separated on another branch.

The K-2-P values between LaolNPV and the MbMNPV/MacoNPV-B/HearMNPV complex for *lef-9*, *polh* and concatenated sequences were greater than 0.05 in all cases (underlined in Table 3 and Table 4). Similarly, the K-2-P values for MacoNPV-A and MacoNPV–B, considered as distinct species, also exceeded 0.05 (dashed line underlined in Table 3 and Table 4). In contrast, K-2-P distances between the two viruses previously considered to be the same species, MbMNPV and MacoNPV-B, were consistent below 0.015 (shown in bold in Table 3 and Table 4). In contrast, when LaolNPV was compared with MacoNPV-A strains the K-2-P distances were intermediate, between 0.05 and 0.015, being of 0.049, 0.028, 0.026 and 0.039 for *lef-8*, *lef-9*, *polh* and the concatenated sequences, respectively (in bold and underlined in Table 3 and Table 4). In this case other characteristics, such as the host range need to be evaluated to establish species status.

**Table 3:** K-2-P values. Pairwise distances of the nucleotide sequences of *lef-9* and *lef-* complete genes. The distances of the virus under study (LaolNPV) to MbMNPV/MacoNPV-B/HearMNPV complex are underlined, the distances to MacoNPV-A strains are in given in bold and underlined. The K-2-P values for MacoNPV-A and MacoNPV-B, two different species, are underlined with a dashed line, while those values between MbMNPV and MacoNPV-B, two viruses of the same viral species, are in bold.

*lef-9 lef-8*	1	2	3	4	5	6	7	8	9	10	11	12	13	14	15	16	17	18	19
1. AcMNPV	-	0.503	0.492	0.044	0.461	0.457	0.487	0483	0.492	0.457	0.468	.0468	0.455	0.470	0.005	0.468	0.488	0.464	0.428
2. AgipNPV	0.455	-	0.190	0.500	0.369	0.282	0.446	0.445	0.353	0.280	0.254	0.254	0.283	0.262	0.503	0.235	0.255	0.213	0.379
3. AgseNPV	0.457	0.193	-	0.482	0.369	0.278	0.468	0.469	0.382	0.276	0.277	0.276	0.275	0.285	0.485	0.220	0.244	0.218	0.388
4. BmMNPV	0.029	0.466	0.457	-	0.450	0.448	0.461	0.458	0.478	0.450	0.458	0.458	0.450	0.456	0.045	0.468	0.486	0.458	0.430
5. ChchSNPV	0.471	0.415	0.415	0.462	-	0.363	0.403	0.406	0.482	0.361	0.355	0.356	0.362	0.357	0.456	0.362	0.367	0.369	0.110
6. HearMNPV	0.458	0.315	0.318	0.456	0.395	-	0.422	0.426	0.421	0.011	0.092	0.092	0.008	0.095	0.545	0.309	0.289	0.288	0.375
7. HearNPV	0.453	0.387	0.395	0.456	0.403	0.401	-	0.005	0.456	0.416	0.411	0.411	0.420	0.413	0.484	0.438	0.422	0.444	0.406
8. HzSNPV	0.448	0.386	0.393	0.452	0.398	0.397	0.010	-	0.456	0.420	0.415	0.415	0.424	0.417	0.480	0.437	0.425	0.443	0.408
9. LdMNPV	0.512	0.329	0.374	0.525	0.484	0.477	0.474	0.468	-	0.419	0.413	0.412	0.420	0.412	0.487	0.400	0.447	0.392	0.477
10. MbMNPV	0.458	0.317	0.317	0.455	0.398	0.006	0.404	0.400	0.478	-	0.091	0.091	**0.005**	0.093	0.454	0.304	0.286	0.284	0.371
11. MacoNPV-A 90/2	0.460	0.302	0.309	0.462	0.396	0.090	0.406	0.403	0.460	0.089	-	0.003	0.089	**0.028**	0.465	0.290	0.295	0.279	0.353
12. MacoNPV A 90/4	0.458	0.302	0.309	0.460	0.396	0.090	0.407	0.403	0.457	0.090	0.003	-	0.089	**0.029**	0.465	0.293	0.297	0.280	0.353
13. MacoNPV-B	0.460	0.313	0.317	0.458	0.398	0.005	0.400	0.398	0.477	**0.004**	0.091	0.091	-	0.092	0.453	0.306	0.285	0.287	0.370
14. LaolNPV	0.464	0.311	0.308	0.468	0.395	0.055	0.392	0.388	0.457	0.051	**0.049**	**0.049**	0.053	-	0.467	0.292	0.295	0.295	0.279
15. RoMNPV	0.040	0.479	0.467	0.061	0.471	0.462	0.463	0.457	0.524	0.463	0.469	0.468	0.466	0.472	-	0.468	0.490	0.464	0.427
16. SeMNPV	0.454	0.252	0.264	0.465	0.424	0.346	0.404	0.400	0.407	0.348	0.328	0.328	0.345	0.336	0.454	-	0.193	0.124	0.366
17. SfMNPV	0.462	0.312	0.308	0.464	0.402	0.357	0.407	0.409	0.471	0.359	0.353	0.353	0.355	0.353	0.458	0.234	-	0.194	0.372
18. SpltNPV	0.462	0.237	0.272	0.479	0.429	0.349	0.400	0.396	0.389	0.354	0.333	0.333	0.351	0.340	0.470	0.132	0.211	-	0.368
19. TnSNPV	0.471	0.406	0.396	0.463	0.127	0.391	0.402	0.400	0.476	0.395	0.391	0.393	0.393	0.397	0.465	0.404	0.386	0.413	-

**Table 4:** K-2-P values. Pairwise distances of the nucleotide sequences of *polh* complete gene and concatenated *polh/lef-8/lef-9* genes. The distances of the virus under study (LaolNPV) to MbMNPV/MacoNPV-B/HearMNPV complex are underlined, the distances to MacoNPV-A strains are in given in bold and underlined. The K-2-P values for MacoNPV-A and MacoNPV-B, two different species, are underlined with a dashed line, while those values between MbMNPV and MacoNPV-B, two viruses of the same viral species, are in bold.

*polh polh/lef-8/lef-9*	1	2	3	4	5	6	7	8	9	10	11	12	13	14	15	16	17	18	19
1. AcMNPV	-	0.243	0.266	0.285	0.211	0.218	0.331	0.337	0.300	0.218	0.199	0.199	0.220	0.198	0.230	0.286	0.236	0.259	0.186
2. AgipNPV	0.432	-	0.163	0.281	0.238	0.196	0.325	0.318	0.308	0.230	0.189	0.189	0.203	0.183	0.294	0.183	0.180	0.169	0.246
3. AgseNPV	0.434	0.188	-	0.303	0.248	0.227	0.325	0.320	0.317	0.237	0.212	0.212	0.233	0.209	0.322	0.204	0.209	0.209	0.269
4. BmMNPV	0.064	0.444	0.440	-	0.304	0.293	0.358	0.353	0.315	0.298	0.302	0.302	0.294	0.292	0.080	0.307	0.304	0.298	0.303
5. ChchSNPV	0.419	0.374	0.374	0.430	-	0.225	0.280	0.281	0.319	0.227	0.231	0.231	0.227	0.230	0.298	0.263	0.213	0.235	0.113
6. HearMNPV	0.415	0.284	0.287	0.424	0.356	-	0.301	0.295	0.300	0.011	0.080	0.080	0.008	0.072	0.304	0.208	0.200	0.219	0.208
7. HearNPV	0.441	0.396	0.401	0.440	0.384	0.390	-	0.012	0.393	0.296	0.315	0.315	0.296	0.317	0.354	0.290	0.308	0.333	0.291
8. HzSNPV	0.437	0.394	0.400	0.435	0.382	0.388	0.009	-	0.390	0.289	0.309	0.309	0.290	0.311	0.347	0.299	0.306	0.340	0.291
9. LdMNPV	0.470	0.334	0.365	0.474	0.458	0.432	0.455	0.452	-	0.308	0.276	0.276	0.304	0.278	0.331	0.334	0.285	0.280	0.300
10. MbMNPV	0.415	0.286	0.288	0.426	0.358	0.008	0.389	0.387	0.433	-	0.089	0.089	**0.003**	0.079	0.304	0.207	0.205	0.227	0.207
11. MacoNPV-A 90/2	0.415	0.269	0.282	0.433	0.357	0.088	0.394	0.392	0.414	0.089	-	0.000	0.089	**0.026**	0.302	0.207	0.205	0.227	0.207
12. MacoNPV A 90/4	0.414	0.269	0.281	0.432	0.357	0.088	0.395	0.393	0.412	0.089	0.002	-	0.089	**0.026**	0.302	0.207	0.187	0.204	0.218
13. MacoNPV-B	0.416	0.285	0.287	0.426	0.358	0.006	0.389	0.387	0.433	**0.004**	0.089	0.089	-	0.080	0.300	0.208	0.206	0.227	0.210
14. LaolNPV	0.417	0.274	0.284	0.433	0.356	0.069	0.387	0.385	0.412	0.067	**0.039**	**0.039**	0.068	-	0.298	0.220	0.187	0.217	0.210
15. RoMNPV	0.055	0.453	0.449	0.058	0.436	0.432	0.448	0.442	0.480	0.432	0.438	0.437	0.432	0.439	-	0.333	0.300	0.305	0.294
16. SeMNPV	0.428	0.235	0.239	0.438	0.380	0.310	0.397	0.395	0.393	0.310	0.294	0.295	0.309	0.302	0.436	-	0.156	0.095	0.272
17. SfMNPV	0.430	0.272	0.272	0.443	0.359	0.308	0.395	0.397	0.431	0.309	0.304	0.305	0.307	0.305	0.441	0.208	-	0.152	0.232
18. SpltNPV	0.428	0.219	0.244	0.442	0.377	0.308	0.403	0.401	0.370	0.311	0.295	0.295	0.310	0.301	0.440	0.123	0.195	-	0.251
19. TnSNPV	0.405	0.373	0.372	0.425	0.120	0.354	0.386	0.386	0.448	0.355	0.350	0.351	0.354	0.354	0.424	0.372	0.356	0.374	-

### Biological characterization

#### LaolNPV has a narrow host range compared to MacoNPV-A

**Fig 5:**
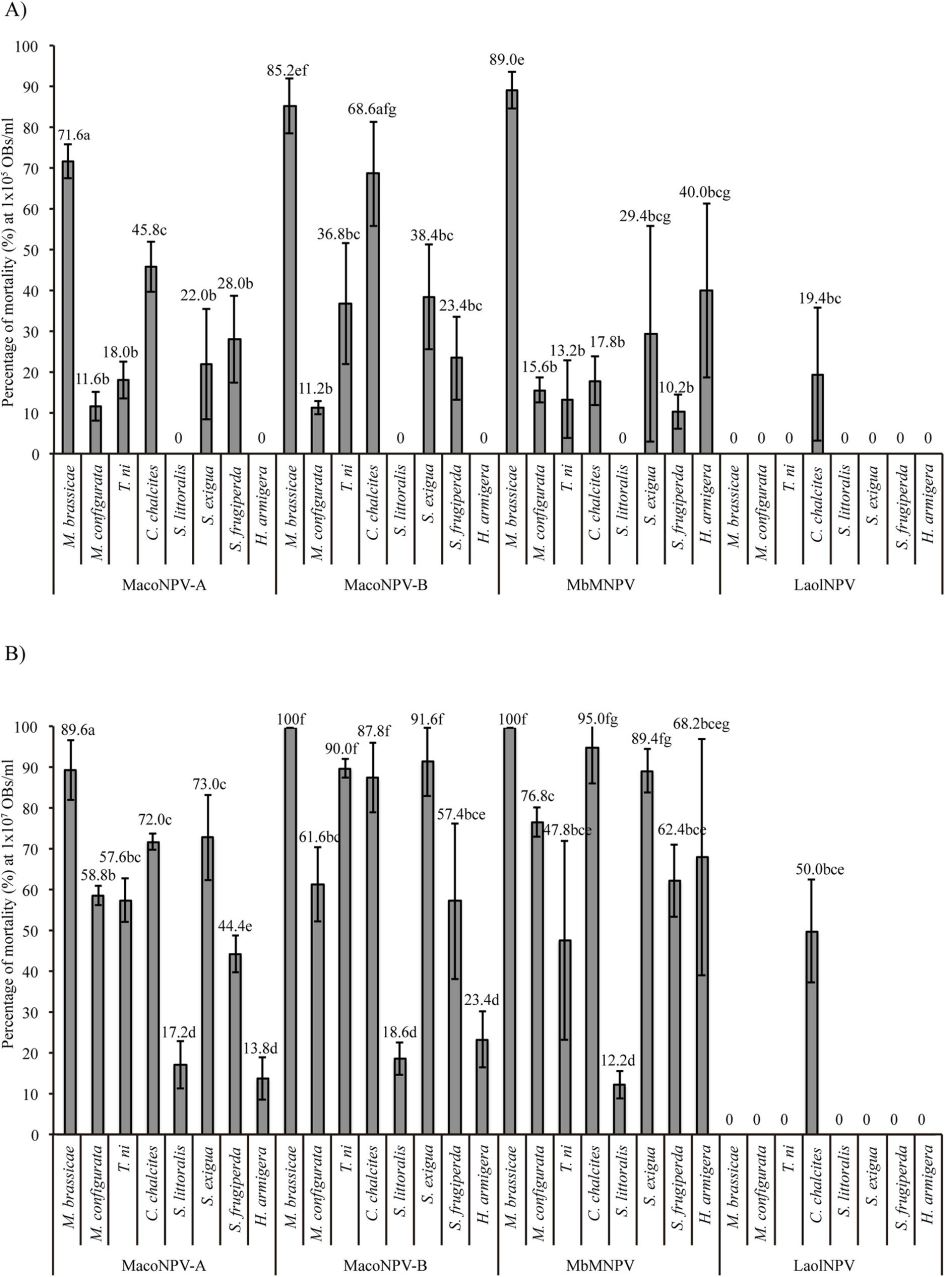
Host range of LaolNPV. Percentage of mortality produced by MacoNPV-A, MacoNPV-B, MbMNPV and LaolNPV OBs inoculated at concentrations of A) 1x10^5^ OBs/ml (≈33 OBs/larva) and B) 1x10^7^ OBs/ml (≈3,300 OBs/larva) against *M*. *brassicae*, *M*. *configurata*, *T*. *ni*, *C*. *chalcites*, *S*. *littoralis*, *S*. *exigua*, *S*. *frugiperda* and *H*. *armigera* larvae. Vertical bars indicate standard errors. Values above bars indicate means. Values followed by different letters are significantly different (Tukey test p<0.05).

The host range of LaolNPV was compared with that of MacoNPV-A, MacoNPV-B and MbMNPV at high (1x10^7^ OBs/ml) (Fig 5A) and moderate (1x10^5^ OBs/ml) (Fig 5B) concentrations in eight lepidopteran species; *M*. *brassicae*, *M*. *configurata*, *T*. *ni*, *C*. *chalcites*, *S*. *littoralis*, *S*. *exigua*, *S*. *frugiperda* and *H*. *armigera*. In all cases dead larvae died due to the inoculated virus as showed in the REN profiles obtained with the OBs from cadavers (data not shown). The REN profile results indicate that cross-contamination did not occur during host range bioassays and OB treatments did not activate sublethal infections by homologous NPVs.

Mortality differed significantly among the insect species at high (F_7,159_ = 114.026; p<0.0001) and moderate (F_7,159_ = 74.955; p<0.0001) OB concentrations. Mortality also varied significantly according to identity of the virus at high (F_3,159_ = 393.261; p<0.0001) and moderate (F_3,159_ = 78.882; p<0.0001) OB concentrations.

MacoNPV-A OBs produced variable mortalities that differed significantly across the different host species (F_7,32_ = 60.253; p<0.0001). The lower concentration of MacoNPV-A OB produced mortalities between 0%, in the least susceptible species such as *S*. *littoralis* or *H*. *armigera*, to 12–72% mortality in the other species. MacoNPV-A was significantly more pathogenic (in terms of the mortality caused by a particular concentration of OBs) to *M*. *brassicae* (72% mortality) than to its homologous species, *M*. *configurata* (12% mortality) (Tukey test p<0.05). At the higher OB concentration MacoNPV-A also caused mortality in *S*. *littoralis* (17%) and *H*. *armigera* (14%), and mortality differed significantly among the different species examined (F_7,32_ = 42.279; p<0.0001). Similarly, at the highest concentration MacoNPV-A was more pathogenic to *M*. *brassicae* (90%) than to *M*. *configurata* (59%) (Tukey test p<0.05), followed by *S*. *exigua* (73%) *C*. *chacites* (72%), and *T*. *ni* (58%) (Tukey test p>0.05) and was least pathogenic to *S*. *frugiperda* (44%).

MacoNPV-B also showed significant variation in pathogenicity to the different species (F_7,32_ = 55.436; p<0.0001). MacoNPV-B was more pathogenic to *M*. *brassicae* (85%), *T*. *ni* (67%), *C*. *chalcites* (69%) and *S*. *exigua* (38%) than to the homologous species *M*. *configurata* (11%) (Tukey test p<0.05). Similarly, at the moderate OB concentration MacoNPV-B did not cause lethal disease in *S*. *littoralis* or *H*. *armigera*, whereas at the higher OB concentration, MacoNPV-B caused mortality in both *S*. *littoralis* (19%) and *H*. *armigera* (23%). MacoNPV-B was markedly more pathogenic to heterologous hosts *M*. *brassicae* (100%), *T*. *ni* (88%), *C*. *chalcites* (88%) and *S*. *exigua* (92%), than to its homologous host *M*. *configurata* (62%).

Although the mortality across species produced by MbMNPV was quite similar to those produced by MacoNPV-A and MacoNPV-B at high and moderate OB concentrations, MbMNPV showed a greater host range as it was pathogenic at the moderate OB concentration to species such as *H*. *armigera*. In general, MbMNPV was more pathogenic to the homologous host *M*. *brassicae* at high (100%) and moderate (89%) concentrations than to the heterologous hosts (Tukey, p<0.05). At the moderate OB concentration MbMNPV produced 100% mortality in *M*. *brassicae* and mortalities lower than 100% in all heterologous species, although these differences were not significant in the case of *C*. *chalcites* or *S*. *exigua* (Tukey, p>0.05).

LaolNPV showed a markedly different host range compared with MacoNPV-A. LaolNPV was not infective to *M*. *brassicae* or *M*. *configurata* even at the high OB concentration. LaolNPV was only pathogenic to *C*. *chalcites* at moderate and high concentrations, which resulted in 19 and 50% mortality, respectively. The host range of LaolNPV was completely different, therefore, to that of MacoNPV-A, indicating that these viruses occupy distinct ecological niches. Regrettably, we were unable to determine the pathogenicity of LaolNPV OBs in *L*. *oleracea* or *A*. *australis* as laboratory or field populations have not been available for testing.

## Discussion

The present study aimed to characterize a novel NPV from diseased larvae of *L*. *oleracea* collected in 2011 from a field of alfalfa near Montpellier, France, although because of difficulties in differentiation of noctuid species in the larval stage we could not be certain of the identity of each and every diseased larva collected.

Restriction endonuclease analyses suggested that the baculovirus under study, LaolNPV, and MacoNPV-A diverged to a greater degree than MbMNPV and MacoNPV-B, that were previously considered to be the same viral species isolated from different hosts [4, 15]. The differences in REN profiles were of a similar magnitude to those found between two different viruses, namely MacoNPV-A and MacoNPV-B. Sucrose density gradients demonstrated that, like MbMNPV, MacoNPV-A and MacoNPV-B [16], LaolNPV is a multiple nucleocapsid virus with a banding pattern quite similar to that of MacoNPV-A, suggesting a similar distribution of multiple nucleocapsids within the ODVs. Additionally, OB and ODV structural polypeptide profiles were also similar to MbMNPV, MacoNPV-A and MacoNPV-B, but with clear differences in the presence and molecular weights of several proteins. These results were consistent with the hypothesis that LaolNPV was closely related to, but distinct from the other NPVs that infect *Mamestra* spp.

Terminal sequencing information revealed that the most homologous NPV to the virus under study was MacoNPV-A 90/2 [14] closely followed by MacoNPV-A 90/4 [16]. The percentage of amino acid sequence identity between LaolNPV and MacoNPV-A was intermediate to that found between strains of MacoNPV-A or MbMNPV/MacoNPV-B, viruses of the same viral species, or between viruses of different species, such as MacoNPV-A and MbMNPV/MacoNPV-B. When comparing with MbMNPV, the identity clearly decreased to a values corresponding to different species. Consequently, it appears that LaolNPV is more similar to the New World MacoNPV-A than to MbMNPV, a species from the Old World (Europe).

Phylogenetic analysis demonstrated that LaolNPV formed a separate branch within a MacoNPV-A clade, with high bootstrap support, as a distinct lineage from MbMNPV, MacoNPV-B and HearMNPV. Additionally, the baculovirus under study differed with respect to the MbMNPV/MacoNPV-B/HaMNPV group with a K-2-P distance greater than 0.05 in all cases, supporting the concept that LaolNPV is phylogenetically different from MbMNPV, HaMNPV or MacoNPV-B. In contrast, the MbMNPV/MacoNPV-B/HaMNPV group had K-2-P distances among the different viruses of less than 0.015.

In many cases the same virus isolated from different hosts has been assigned different names. This was the case with *Anagrapha falcifera* NPV (AnfaNPV) and *Rachioplusia ou* MNPV (RoMNPV) [43, 51] and HearSNPV and HzSNPV [40]. Alternatively, different viruses isolated from the same species have received the same name [52]. Although both MacoNPV-A and MacoNPV-B were isolated from the same host and their genomes are closely related, MacoNPV-B represents a separately evolving virus from MacoNPV-A, as the K-2-P distances estimated in the present study were consistently higher than 0.05 (0.088), and exceed the genetic distance between RoMNPV and BmNPV (0.055), which are also thought to be different species [4, 43]. MacoNPV-A is much more prevalent and infective to *M*. *configurata* populations than MacoNPV-B, however MacoNPV-B has higher pathogenicity to a wider range of host species. It is likely that there are alternative hosts for MacoNPV-B, so that infections in *M*. *configurata* are only commonly observed during outbreak years of high population density. It is intriguing that these two viruses could evolve divergently while infecting the same host, *M*. *configurata* [4, 14, 15]. This type of situation highlights the present drawbacks in baculovirus nomenclature as virus names are based on the host from which the virus was first isolated. However, it is also clear that host range can prove to be an informative aspect among the ecological characteristics that contribute to the baculovirus species definition criteria.

Baculovirus isolates from separate populations of a particular host species, or from closely related species, often differ in pathogenicity and the origin of a particular NPV is not a useful predictor of its virulence in a specific host, although local isolates tend to be more effective in controlling local insect populations than geographically distant isolates [6, 13, 21, 53]. Surprisingly, although the baculovirus under study was phylogenetically close to MacoNPV-A, LaolNPV showed particular host range differences and was not pathogenic to *M*. *brassicae* or *M*. *configurata*, even at moderately high OB concentrations. In contrast, MacoNPV-A produced mortality across a variety of species tested. Therefore the effective host range of MacoNPV-A appears to be notably broader than that of LaolNPV. This suggests that these viruses occupy different ecological niches, and therefore, in combination with the phylogenetic analyses, are likely to represent different species. Surprisingly, the divergence in pathogenicity between viruses within related insect populations was also observed with the MacoNPV viruses. MacoNPV-B was more pathogenic than MacoNPV-A for several species. Additionally both MacoNPV-A and -B viruses were more pathogenic to *M*. *brassicae* than to the homologous host, *M*. *configurata*. This surprised us, as NPVs tend to be more pathogenic to homologous hosts rather than heterologous species [21, 54, 55]. However, in the present study we observed that MbMNPV was more pathogenic to *M*. *configurata* than MacoNPV-A or -B, which was also reported by Erlandson [6]. MbMNPV and MacoNPV-B represent variants of the same viral species, with distributions that include southern Canada and Europe [15]. Both viruses infect *M*. *configurata*, and as MbMNPV is common in Europe, it is possible that MacoNPV-B-like viruses may be found worldwide.

We conclude that *Lacanobia oleracea nucleopolyhedrovirus* (LaolNPV) should be considered as a new species of multiple nucleocapsid NPV in the *Alphabaculovirus* genus on the basis of REN profiles, gene sequence features and host range properties, in addition to the original insect host species and geographical point of isolation. Future studies should examine intraspecific variation in LaolNPV and establish the dose or concentration mortality response of this virus in its homologous host and sympatric noctuid species, such as *Agrochola lychnidis*, that are present in the alfalfa agroecosystem of southern Europe. Such information could prove valuable in evaluating the virus’ potential as a biological insecticide for control of *L*. *oleracea* and related noctuid pests in this region.
